# Glucose-6-phosphate dehydrogenase deficiency and the risk of developing systemic lupus erythematosus

**DOI:** 10.3389/fmed.2026.1758860

**Published:** 2026-04-16

**Authors:** Xiao-hua Yu, Yue-feng Yao, Bai-ru Lai, Yan-bin Cao, Jian-lian Liang, Zhen Wang, Zhi-xiao Chen, Bao-ying Chen, Yu-chan Huang, Yan-qing Zeng, Li-ye Yang

**Affiliations:** 1 Precision Medical Lab Center, People's Hospital of Yangjiang, Yangjiang, Guangdong, China; 2 Department of Rheumatology and Immunology, People's Hospital of Yangjiang, Yangjiang, Guangdong, China; 3 Blood Transfusion Department, People's Hospital of Yangjiang, Yangjiang, Guangdong, China

**Keywords:** gene mutation, glucose-6-phosphate dehydrogenase, glucose-6-phosphate dehydrogenase (G6PD) deficiency, risk factor, systemic lupus erythematosus

## Abstract

**Objective:**

To explore the relationship between glucose-6-phosphate dehydrogenase (G6PD) deficiency and the risk of developing systemic lupus erythematosus (SLE).

**Methods:**

A case-control study was conducted including 516 female SLE patients (SLE group) and 491 age-matched healthy females (control group) from August 2023 to August 2024. G6PD enzymatic activity was detected via the G6PD/6PGD ratio method, and G6PD gene mutations were identified by PCR combined with flow hybridization; 21 G6PD-deficient samples underwent whole-exome sequencing (WES) for genotype verification. The two groups were stratified by enzymatic status, and differences in enzymatic ratios, gene mutation rates and mutation types were compared using rank-sum test and Pearson's chi-square test with SPSS 25.0.

**Results:**

The G6PD enzymatic deficiency rates were 9.69% (50/516) in the SLE group and 10.79% (53/491) in the control group (*P* > 0.05), with no significant differences in age or enzymatic ratios between groups (*P* > 0.05). Gene mutation rates showed no intergroup differences in either enzymatic deficiency (84.00% vs. 88.68%) or normal subgroups (8.37% vs. 10.05%, all *P* > 0.05). WES identified no novel variants, with c.1376G > T and c.1388G > A as the most common mutations and similar frequencies in both groups (*P* > 0.05).

**Conclusion:**

G6PD deficiency does not correlate with the risk of developing systemic lupus erythematosus.

**Funding project:**

Yangjiang High-level Key Medical and Health Research Project (2023001).

## Introduction

Glucose-6-phosphate Dehydrogenase (G6PD) Deficiency is an X-linked incomplete dominant genetic disorder. The G6PD gene is located at Xq28, with a full length of 20,114 bp, consisting of 13 exons and 12 introns, coding for a polypeptide chain made up of 515 amino acids (1). The average prevalence of G6PD deficiency is 8% in malaria-endemic countries and 5.3% in countries currently planning malaria elimination (2). High prevalence rates are observed in sub-Saharan Africa, Asia, the Middle East, Latin America, and the Mediterranean region (3). It is estimated that around 400 million people worldwide carry mutations related to enzyme deficiency, showing significant racial and geographical differences (4, 5). The prevalence of G6PD deficiency varies significantly between northern and southern China, with a nationwide prevalence of 2.10% (6). In Yangjiang of Guangdong province, the prevalence of G6PD deficiency is 7.54% (250/3314) in males (7).

Systemic Lupus Erythematosus (SLE) is an acute or chronic autoimmune disease characterized by the formation of pathogenic autoantibodies and immune complexes that mediate damage to multiple organs, predominantly affecting women of childbearing age, and displaying significant heterogeneity. Previous studies have indicated that individuals with G6PD deficiency are more likely to develop systemic lupus erythematosus compared to those without G6PD deficiency (8). However, there are currently no related studies in China regarding the increased risk of systemic lupus erythematosus in individuals with G6PD deficiency. Guangdong Province in China is a high-incidence area for G6PD deficiency (9), and this study aims to explore the risk of developing systemic lupus erythematosus in the G6PD-deficient population in the Yangjiang area of western Guangdong Province.

## Objects and methods

### Objects

Using the sample size estimation formula for two independent proportions [*n* = 2(Z_α/2_ + Z_β_)^2^/d^2^] for this case-control study, the key statistical parameters were set based on the international standards for autoimmune disease association research and preliminary epidemiological data of G6PD deficiency in southern China: two-sided α (type I error) = 0.05 (Z_α/2_ = 1.96), β (type II error) = 0.20 (Z_β_ = 0.84, corresponding to 80% statistical power), and allowable absolute error d = 0.20. Based on the above parameters, the minimum required sample size for a single group was calculated to be 392 cases. To account for potential sample loss, test failure and incomplete follow-up during the study, we appropriately expanded the sample size and finally enrolled 516 female patients diagnosed with systemic lupus erythematosus (SLE) in accordance with international clinical guidelines (10). These patients were admitted to the People's Hospital of Yangjiang, Guangdong Province, China, from August 2023 to August 2024, and constituted the SLE group. Meanwhile, 491 age-matched, randomly selected Han Chinese females from southern China who underwent routine health check-ups at our hospital were recruited as the control group, with no racial or ethnic heterogeneity among all participants. General demographic information including gender (all participants were female) and age was collected for all subjects in both the SLE and control groups. All enrolled individuals underwent G6PD/6PGD enzymatic ratio detection and G6PD gene mutation analysis. This study was approved by the Ethics Review Committee of the People's Hospital of Yangjiang (Approval No.: 20230003), and all participants provided written informed consent prior to enrollment.

### Methods

#### Sample collection

Venous blood (1–2 mL) was drawn from study subjects using EDTA-K_2_ anticoagulant vacuum tubes, stored at 2–8°C, and tested for G6PD/6PGD ratio and G6PD gene mutations within 3 days.

#### G6PD/6PGD ratio detection and determination criteria

The collected anti-coagulated blood was mixed, and 20 μL was added to 1 mL of the G6PD detection kit (ratio method) solution. After thorough mixing for 2 min, the detection was performed using a BIOELAB fully automated biochemical analyzer. According to the kit instructions, if G6PD/6PGD ≥ 1.0 (for adults), it was determined as G6PD normal (enzymatically normal); if G6PD/6PGD < 1.0 (for adults), it was determined as G6PD deficiency (enzymatically deficient) (11).

#### G6PD gene mutation detection and result interpretation

G6PD gene mutation detection was performed for 516 SLE cases and 491 controls using PCR combined with flow hybridization (dot blot hybridization, DBH). The specific steps are as follows: 1–2 mL of the collected anticoagulated blood was used to extract genomic DNA using a kit from Hybribio. Gene amplification was conducted using a PCR amplification instrument, and the amplification products were subjected to flow hybridization on a medical nucleic acid molecular hybridization instrument. Result interpretation was performed according to the instructions of the G6PD gene detection kit, testing for 13 G6PD gene mutation sites: c.95A>G, c.392G>T, c.487G>A, c.592C>T, c.871G>A, c.1024C>T, c.1360C>T, c.1311C>T, c.1376G>T, c.493A>G, c.592G>T, c.1004C > T, and c.1388G>A (7).

#### Whole exome sequencing analysis

Whole exome sequencing analysis was performed on 21 samples with G6PD deficiency, using the Agilent SureSelect XT Human All Exome V6 kit (Agilent Technologies, Santa Clara, USA) for NGS sequencing. Novogene Bioinformatics Technology Co., Ltd. (Beijing, China) performed genomic DNA sequencing using the Illumina NovaSeq 6000 platform (Illumina, San Diego, California, USA), generating 150 bp paired-end reads with a minimum coverage of 99% (sequencing depth of 200x). The analysis covered the complete coding region, splice junctions (10 bp upstream and 10 bp downstream), and regulatory regions of the target genes (12). We performed whole-exome sequencing (WES) on 21 patients with dual purposes: (1) to validate the accuracy of our blot hybridization-based genotyping results, and (2) to investigate potential missed mutations in patients exhibiting G6PD/6PGD ratios < 1.0 without identified pathogenic variants.

#### Statistical analysis methods

Statistical analysis was performed using SPSS version 25.0. Measurement data were expressed as median (interquartile range) M (IQR). For comparisons between groups with non-normally distributed data, the rank-sum test was used. Count data were expressed as percentages (%), and comparisons between groups were made using Pearson's chi-square (X^2^) test, with *P* < 0.05 considered statistically significant.

## Results

### G6PD/6PGD ratio

A total of 1,007 subjects were included in the study, with 516 in the SLE group and 491 in the control group. Both groups were divided into enzymatically deficient and enzymatically normal subgroups based on the results of the G6PD/6PGD enzymatic ratio (cut-off value of 1). There were no statistically significant differences in age and enzymatic ratios between the SLE group and the control group in both the enzymatically deficient and enzymatically normal subgroups (*P* > 0.05) (Table 1).

**Table 1:** Comparison of general data between SLE group and control group.

	Total (*n* = 1,007)	SLE Group (*n* = 516)	Control group (*n* = 491)	*Z*	*P*
Enzymatic deficiency					
Age (years)	39 (32, 46) (*n* = 103)	42.5 (33, 52) (*n* = 50)	36 (32, 44) (*n* = 53)	1.91	0.056
15.6-7.4,-14.3498ptG6PD/6PGD enzymatic ratio	0.71 (0.46, 0.86) (*n* = 103)	0.68 (0.41, 0.90) (*n* = 50)	0.71 (0.55, 0.82) (*n* = 53)	0.54	0.593
Enzymatic normal					
Age (years)	40 (31, 50) (*n* = 904)	40 (31, 51) (*n* = 466)	40 (31, 50) (*n* = 438)	1	0.317
G6PD/6PGD enzymatic ratio	1.58 (1.43, 1.75) (*n* = 904)	1.58 (1.44, 1.73) (*n* = 466)	1.58 (1.41, 1.77) (*n* = 438)	0.43	0.60

### G6PD gene testing results

Gene mutation testing (reverse dot blot hybridization) was completed for 1,007 patients in both groups. In the SLE enzymatically deficient group, the number of positive results from dot blot hybridization was 42 (42/50), while in the control group, it was 47 (47/53). In the SLE enzymatically normal group, there were 39 positive results (39/466), and in the control group, 44 positive results (44/438) were observed (Table 2).

**Table 2:** Genetic testing results between SLE group and control group.

	Total (*n* = 1,007)	SLE group (*n* = 516)	Control group (*n* = 491)
Enzymatic deficiency	103 (10.23%)	50 (9.69%)	53 (10.79%)
Positive dot blot hybridization	89 (86.40%)	42 (84.00%)	47 (88.68%)
Negative dot blot hybridization	14 (13.60%)	8 (16.00%)	6 (11.32%)
Enzymatic normal	904 (89.77%)	466 (90.31%)	438 (89.21%)
Positive dot blot hybridization	83 (9.18%)	39 (8.37%)	44 (10.05%)
Negative dot blot hybridization	821 (90.82%)	427 (91.63%)	394 (90.95%)

### Whole exome sequencing analysis

Among the SLE group, there were 50 cases with enzymatic deficiency, of which 8 were negative by DBH, and among the control group, there were 53 cases with enzymatic deficiency, with 6 having negative results for genetic testing. Fourteen cases (G6PD/6PGD enzymatic ratio in 12 cases were 0.8–1.0, 2 cases were lower than 0.8) from both groups that were enzymatically deficient but negative by DBH underwent whole exome sequencing analysis, and the results were negative. Five cases with the mutation c.1376G>T and two cases with c.1388G>A identified with DBH were subsequently validated by whole exome sequencing, and these results were consistent with those obtained from PCR combined with flow hybridization (Table 3).

**Table 3:** The results of whole exome sequencing analysis.

	Dot blot results (*n* = 21)	Whole exome sequencing results (*n* = 21)
SLE group (*n* = 13)		
Negative (*n* = 8)	Negative (*n* = 8)	Negative (*n* = 8)
1376M (*n* = 5)	1376M (*n* = 5)	1376M (*n* = 5)
Control group (*n* = 8)		
Negative (*n* = 6)	Negative (*n* = 6)	Negative (*n* = 6)
1388M (*n* = 2)	1388M (*n* = 2)	1388M (*n* = 2)

### G6PD enzymatic deficiency rate and gene mutation rate

In the SLE group, there were 50 cases of enzymatic deficiency (50/516) and 466 cases of enzymatic normal (466/516), with G6PD gene mutations in 42 cases (42/50) and 39 cases (39/466), respectively. In the control group, there were 53 cases of enzymatic deficiency (53/491) and 438 cases of enzymatic normal (438/491), with G6PD gene mutations in 47 cases (47/53) and 44 cases (44/438), respectively. There were no statistically significant differences in the rates of enzymatic deficiency and gene mutation rates between the two groups (*P* > 0.05), nor in the mutation rates of the enzymatically normal group or the overall gene mutation rates (*P* > 0.05) (Table 4).

**Table 4:** Enzymatic deficiency rates and gene mutation rates in SLE group and control group.

	SLE group (*n* = 516)	Control group (*n* = 491)	*X* ^2^	*P*
Enzymatic deficiency				
Enzymatic deficiency rate	50/516 (9.69%)	53/491 (10.79%)	0.33	0.563
Gene mutation rate for enzymatic deficiency	42/50 (84.00%)	47/53 (88.68%)	0.48	0.489
Enzymatically normal				
Gene mutation rate	39/466 (8.37%)	44/438 (10.05%)	0.76	0.38
Total gene mutation rate	81/516 (15.70%)	91/491 (18.53%)	1.43	0.23

### Types of G6PD gene mutations

All 1,007 patients underwent G6PD gene mutation testing, identifying a total of 11 types of gene mutations: c.95A>G, c.392G>T, c.1024C>T, c.1376G>T, c.1388G>A, c.871G>A, c.1360C>T, c.1311C>T, c.1388G>A/c.1004C>A, c.1376G>T/c.1388G>A, and c.95A>G/c.1024C>T. Among these, c.1376G>T and c.1388G>A were the most common. The remaining mutations that are not included in the Table 5 are c.1311C>T (benign variant) and negative results (Table 5).

**Table 5:** The types of gene mutations in SLE group and control group.

Gene mutation type	95M	392M	1024M	1376M	1388M	871M	1360M	1388M/ 1004M	1376M/ 1388M	95M/ 1024M	Total
SLE group											
Enzymatic deficiency	2	1	1	14	17	6	0	1	0	0	42
Enzymatically normal	6	4	2	15	9	3	0	0	0	0	39
Total	8	5	3	29	26	9	0	1	0	0	81
Control group											
Enzymatic deficiency	9	3	2	9	20	2	0	0	1	1	47
Enzymatically normal	9	5	2	11	15	2	0	0	0	0	44
Total	18	8	4	20	35	4	0	0	1	1	91

### Comparison of mutation frequencies of c.1376 and c.1388 between groups

In the gene testing results, c.1376G>T and c.1388G>A were the most common mutations. The comparison of mutation frequencies for c.1376G>T and c.1388G>A between the SLE group and the control group in both enzymatically deficient and enzymatically normal subgroups showed no statistically significant differences (*P* > 0.05) (Table 6).

**Table 6:** The comparison of mutation frequencies of 1,376 and 1,388 between two groups.

	Genotype	SLE group	Control group	*X* ^2^	*P*
Enzymatic deficiency					
	1376M	14/42	9/47	2.33	0.127
	1388M	17/42	20/47	0.04	0.843
Enzymatically normal					
	1376M	15/39	11/44	1.74	0.19
	1388M	9/39	15/44	1.22	0.27

## Discussion

Glucose-6-phosphate dehydrogenase (G6PD) is a key enzyme in the pentose phosphate pathway (PPP), responsible for generating reduced nicotinamide adenine dinucleotide phosphate (NADPH) (13). NADPH plays an important antioxidant role in cells, helping to maintain redox balance. G6PD deficiency leads to reduced NADPH production, which decreases the cell's resistance to oxidative stress and increases the risk of cellular damage (14). G6PD deficiency makes red blood cells more susceptible to oxidative damage in high-oxygen environments, potentially leading to hemolytic anemia. This type of anemia often worsens under oxidative stress triggered by infections, certain medications, or foods (such as fava beans) (4). In red blood cells with G6PD deficiency, glutathione (GSH) levels rapidly decline, accompanied by the accumulation of oxidized glutathione (GSSG). This depletion of GSH can lead to a cellular energy crisis and trigger ineffective activation of AMP-activated protein kinase (AMPK), further exacerbating metabolic dysregulation. G6PD deficiency is the most common cause of congenital hemolytic anemia, affecting approximately 4.9% of the global population (15). It is also the most common enzymatic X-linked human disease, primarily affecting red blood cells (16).

The pathogenesis of systemic lupus erythematosus (SLE) is complex and involves multiple factors, including genetic, environmental, and immune system interactions. The main aspects of SLE pathogenesis are as follows: Genetic Susceptibility: Recent genome-wide association studies (GWAS) have identified over 90 susceptibility loci associated with SLE, many of which contain single nucleotide polymorphisms (SNPs) that affect the clearance of immune complexes, lymphocyte signaling, and type I interferon (IFN-I) signaling pathways. Some rare monogenic forms of SLE have also been reported, indicating that genetic factors play a crucial role in disease development (17). Environmental Factors: Ultraviolet (UVB) radiation can induce reactive oxygen species and upregulate the expression of pro-inflammatory cytokines such as interferon-alpha (IFN-α), interleukin-1 (IL-1), interleukin-6 (IL-6), and tumor necrosis factor-alpha (TNF-α), leading to skin damage and exacerbation of the disease. Additionally, UVB exposure induces DNA hypomethylation in CD4^+^T cells, promoting the production of autoreactive cells and autoantibodies (18). Viral infections (such as Epstein-Barr virus) have been associated with the onset of SLE, potentially promoting autoimmune responses through molecular mimicry mechanisms (19). Certain medications (such as hydroxyethyl-aminobenzoic acid and isoniazid) can induce drug-induced lupus, suggesting interactions between drug metabolism and immune responses. Abnormalities in the Immune System: In genetically susceptible individuals, environmental factors can lead to the loss of immune tolerance, activating autoreactive B cells and T cells that produce autoantibodies (such as anti-double-stranded DNA antibodies). Therefore, exposure to environmental factors like UVB radiation, viral infections, and drugs can trigger the loss of immune tolerance in genetically susceptible individuals, leading to abnormal activation of autoimmunity and systemic immune responses (20).

From the above pathogenesis, there is no direct correlation between G6PD deficiency and SLE. Clinically, G6PD deficiency primarily leads to hemolysis, while SLE mainly triggers systemic immune responses. In the study by Fujii J et al. (21), it was pointed out that insufficient supply of NADPH leads to a state of oxidative stress in cells, increasing the production of reactive oxygen species (ROS). ROS are generated and accumulate in red blood cells (RBCs), potentially causing oxidative modifications of membrane proteins. These oxidatively modified proteins are recognized by the immune system as foreign antigens, triggering autoimmune responses. Furthermore, this study used animal models to investigate the pathogenesis of SLE and found no abnormalities in the G6PD gene in New Zealand black mice, although these mice exhibited higher levels of ROS during aging (21). Therefore, the oxidative stress and associated autoimmune responses in New Zealand black mice may be related to other factors rather than directly caused by defects in the G6PD gene.

Israel et al. (8) conducted a retrospective study involving 7,473 individuals with G6PD deficiency and 29,892 individuals without G6PD deficiency over a 20-year period in Israel. They observed a significantly increased incidence of autoimmune diseases, including SLE, in individuals with G6PD deficiency compared to those without (8). However, our study did not find this phenomenon. Another study suggests that the frequency of G6PD deficiency in rheumatic population is similar to that of the general population (22). The methodology of Israel et al. (8) differs from that of our study; they conducted a retrospective study using diagnostic data provided by medical institutions, while our study performed enzymatic ratio testing and G6PD gene mutation testing on all included subjects. Additionally, to verify the accuracy of the G6PD gene mutation testing results, we selected 21 patients for whole exome sequencing, and the results were consistent with those from the reverse dot blot hybridization G6PD gene mutation testing.

From an epidemiological perspective, the prevalence of SLE varies among different regions and ethnicities. High-risk populations for SLE are primarily concentrated in the United Arab Emirates (Asia), Barbados (North America), and Brazil (South America) (23). The prevalence of SLE in China is 47.53 per 100,000 (24). In contrast, populations with a high prevalence of G6PD deficiency are mainly found in Africa, Asia, the Middle East, Latin America, and the Mediterranean region (25). The high-risk areas for SLE do not completely overlap with those for G6PD deficiency.

In this study, when comparing the SLE group with the control group, there were no statistically significant differences in the enzyme levels, enzyme deficiency rates, or gene mutation rates between systemic lupus erythematosus patients and the control group. This study found that G6PD deficiency is not associated with the risk of developing systemic lupus erythematosus, which is inconsistent with the conclusions of Israel et al. (8), who reported that patients with G6PD deficiency are more likely to develop systemic lupus erythematosus. The observed discrepancy may be attributed to distinct G6PD genotype distributions between Israeli and southern Chinese populations (6, 8). Globally, over 200 types of G6PD gene mutations have been identified, with geographical differences (26). In Thailand, the most common mutations are c.871G>A and c.487G>A (27), while in Myanmar and Pakistan, c.563C>T is the most prevalent (28, 29). In Spain, the most common mutations are c.376A>G and c.202A> G (30), and in the Nigerian population, c.202A>G and c.376A>G are common (31). Among the 11 types of G6PD mutations detected in our study, c.1376G>T and c.1388G>A were the most common, consistent with the common mutation types in the Chinese population (6). Moreover, in our study, the mutation frequencies of c.1376G>T and c.1388G>T were similar between the SLE group and the control group.

This study has some limitations. Firstly, since SLE is more common in females, our study included only female patients. Secondly, the sample size is relatively small, being limited to patients from a single tertiary hospital, which may introduce regional bias.

In summary, our study indicates that G6PD deficiency is not associated with the risk of developing systemic lupus erythematosus. Our findings are inconsistent with the results from the Israeli population (8), which may be related to population differences. We will further investigate the underlying mechanisms in future research.

## Data Availability

The raw data supporting the conclusions of this article will be made available by the authors, without undue reservation.
